# AQP1 and AQP4 Contribution to Cerebrospinal Fluid Homeostasis

**DOI:** 10.3390/cells8020197

**Published:** 2019-02-24

**Authors:** José Luis Trillo-Contreras, Juan José Toledo-Aral, Miriam Echevarría, Javier Villadiego

**Affiliations:** 1 Instituto de Biomedicina de Sevilla-IBiS, Hospital Universitario Virgen del Rocío/CSIC/Universidad de Sevilla, Sevilla 41013, Spain; jtrillo@us.es (J.L.T.-C.); juanjo@us.es (J.J.T.-A.); 2 Departamento de Fisiología Médica y Biofísica, Universidad de Sevilla, Sevilla 41009, Spain; 3 Centro de Investigación Biomédica en Red sobre Enfermedades Neurodegenerativas (CIBERNED), Madrid 28029, Spain

**Keywords:** aquaporins, AQP1, AQP4, cerebrospinal fluid, astrocytes, choroid plexus, cerebral ventricles

## Abstract

Aquaporin 1 (AQP1), expressed in epithelial cells of the choroid plexus, and aquaporin 4 (AQP4) present in ependymal cells and glia limitants have been proposed to play a significant role in cerebrospinal fluid (CSF) production and homeostasis. However, the specific contribution of each water channel to these functions remains unknown, being a subject of debate during the last years. Here, we analyzed in detail how AQP1 and AQP4 participate in different aspects of the CSF homeostasis such as the load and drainage of ventricles, and further explored if these proteins play a role in the ventricular compliance. To do that, we carried out records of intraventricular pressure and CSF outflow, and evaluated ventricular volume by magnetic resonance imaging in AQP1^−/−^, AQP4^−/−^, double AQP1^−/−^-AQP4^−/−^ knock out and wild type mice controls. The analysis performed clearly showed that both AQPs have a significant participation in the CSF production, and additionally revealed that the double AQP1-AQP4 mutation alters the CSF drainage and the ventricular compliance. The data reported here indicate a significant extra-choroidal CSF formation mediated by AQP4, supporting the idea of an important and constant CSF production/absorption process, sustained by efflux/influx of water between brain capillaries and interstitial fluid. Moreover, our results suggest the participation of AQPs in structural functions also related with CSF homeostasis such as the distensibility capacity of the ventricular system.

## 1. Introduction

Cerebrospinal fluid (CSF) and interstitial fluid (ISF) are crucial components of the cerebral extracellular fluid, being in dynamic equilibrium and bathing neurons and glial cells. The continuous and strictly regulated interchange between CSF and ISF provides a stable brain volume and fluid content of ions and other solutes for neuronal and glial function [1,2,3]. CSF is produced by modification of the circulating plasma and fills the brain ventricles and the subarachnoid space. In addition to its mechanical function protecting the central nervous system (CNS), CSF represents a fundamental pathway for waste clearance in neural tissue. According to the “classic view” of CSF homeostasis, CSF is mainly produced by the choroid plexus, circulating unidirectionally through the ventricular system and is drained via arachnoid granulations into the meningeal sinus or via lymphatic vessels of the nasal mucosa after crossing the cribriform plate [1,4]. However, recently acquired knowledge has modified this “classic view” to a new scenario where: (i) there is an important extra-choroidal production of CSF by fluid interchange between brain capillaries and ISF [5]; (ii) a significant paravascular flow of CSF/ISF could occur in the brain parenchyma [6,7,8,9]; and (iii) CSF is largely drained by the meningeal lymphatic system [3,10,11]. Alterations on the CSF homeostasis have been strongly associated with the pathophysiology of different CNS pathologies such as hydrocephalus, cerebral edema or ischemia [2,5,12,13]. Moreover, recent findings suggest that a diminished CSF production or drainage accelerates the decay of brain function during aging or aging-associated neurodegenerative diseases [13,14,15,16].

Cerebral aquaporins (AQPs), mainly AQP1 and AQP4, have been proposed to play a relevant function on the CSF and ISF homeostasis. Based on their expression pattern, AQP1 expressed only in the choroid plexus epithelial cells and AQP4 in ependymal cells and glia limitants, including pericapillary astrocyte foot processes, a simplistic view of their function associates AQP1 to CSF production and AQP4 to CSF/ISF exchange and absorption [17,18]. In accord with the classic CSF circulation theory, AQP1^−/−^ mice showed decreased CSF production and intraventricular pressure, suggesting a prominent role of the AQP1 on CSF formation [19]. However, a study performed in AQP1^−/−^ and AQP4^−/−^ mice concluded that water influx to the CSF is mediated by AQP4, and not by AQP1, indicating an important role of AQP4 on CSF formation [20]. This finding supports the so-called “Bulat–Klarica–Oreskovic hypothesis”, which proposes an important and constant CSF formation widely distributed in the brain as consequence of water exchange between brain capillaries and ISF [5]. Furthermore, the description by Iliff et al. of a “glymphatic” system that regulates the CSF/ISF exchange through an AQP4-facilitated paravascular flow between peri-arterial and peri-venous exchange [6,8,21] has provided additional evidence about the important role of AQP4 on CSF homeostasis. Even though the exact contribution of AQP4 to the so-called “glymphatic” system is currently under debate [2,22,23], there is a broad consensus about AQP1 and AQP4 being fundamental mediators on the CSF brain fluid regulation. Notwithstanding, the precise contribution of AQP1 and AQP4 to the different aspects of the CSF/ISF homeostasis, such as CSF production and drainage, CSF/ISF exchange or the regulation of the ventricular system, is still unknown.

In this work, we study the specific contribution of the principal cerebral AQPs, AQP1 and AQP4, to fundamental properties of the CSF homeostasis, such as CSF formation and outflow, and distensibility of the ventricular system. To this end, we performed a comparative analysis of total ventricular volume, intraventricular pressure (IVP), CSF outflow rate and ventricular compliance in wild type (wt), AQP1^−/−^, AQP4^−/−^ and double-AQP1^−/−^-AQP4^−/−^ knock out (double-AQP^−/−^KO) mice. The obtained results indicate that AQP4 has an important role on the CSF formation, which is comparable to the choroid plexus AQP1 dependent CSF production. That support a significant extra-choroidal CSF formation as predicted by the “Bulat-Klarica-Oreskovic hypothesis. Moreover, and for the first time remarked, our in vivo analysis of the ventricular compliance suggests that lack of AQP1 and AQP4 expression reduces the CSF drainage and the distensibility of the cerebral ventricles.

## 2. Material and Methods

### 2.1. Animal Care

Male AQP1^−/−^, AQP4^−/−^, double-AQP^−/−^ KO mice and wt littermates were housed at 22 ± 1 °C in a 12 h light/dark cycle, with ad libitum access to food and water. Animals were 2–6 months old for all experimental procedures. Both AQP1^−/−^ and AQP4^−/−^ animals had a C57BL/6 genetic background, obtained by more than nine generations of outcrossing. Double-AQP^−/−^ KO mice were generated in our laboratory by crossing the AQP1^−/−^ and AQP4^−/−^ mice colonies used before [13]. Previously, Song et al. [24] described the generation of a double AQP1/4-KO animal using a similar breeding strategy as used here. The general characterization of double-AQP^−/−^ KO mice is shown in Figure S1. AQP1 and AQP4 mutant alleles were genotyped as previously described [25,26,27]. For sacrifice, animals received terminal anesthesia, by combination of 100 mg/kg ketamine (Pfizer, New York, NY, USA) and 10 mg/kg xylazine (Bayer, Leverkusen, Germany).

All experiments were carried out in accordance with the European Directive 2010/63/EU and the Spanish RD/53/2013 for the protection of animals used for scientific purposes; and all animal procedures counted with the approval of the Animal Research Committee of the Virgen del Rocío University Hospital (26/01/2017/017; University of Seville, Seville, Spain).

### 2.2. Histological Analyses

Mice were perfused with 50 mL of PBS (Sigma, St. Louis, MO, USA) and 50 mL of 4% paraformaldehyde (Sigma) in phosphate buffer solution (PBS). Brains were extracted immediately and fixed at 4 °C with 4% paraformaldehyde in PBS. For the analysis of the choroid plexus, tissue was dissected from the brains under a stereoscopic binocular microscope (SZX16; Olympus, Tokio, Japan) and immunofluorescence was performed as described before [13]. For brain analysis, tissue was processed and cut in a cryostat as previously described [28,29].

Glial fibrillary acid protein (GFAP), AQP1 and AQP4 immunofluorescence analyses were performed as described previously [13,30], using monoclonal anti-GFAP (1:300; G3893, Sigma), polyclonal AQP1 (1:500; ab15080, Abcam, Cambridge, UK) and polyclonal AQP4 (1:100; AQP41-A, α-Diagnostic, San Antonio, TX, USA). Anti-mouse IgG conjugated with Alexa Fluor488 or anti-rabbit IgG conjugated with Alexa Fluor568 (1:400; A21202 and A11036, respectively, Invitrogen, Carlsbad, CA, USA) were used as secondary antibodies. 4′,6-Diamidino-2-phenylindole (DAPI; 1:1000; Sigma) was used for the nuclei staining. Tissue sections were mounted with Dako fluorescence mounting medium (Dako, Santa Clara, CA, USA). Confocal images were obtained using a A1R^+^ confocal microscope (Nikon, Tokio, Japan).

### 2.3. Magnetic Resonance Imaging

Magnetic resonance imaging (MRI) was assessed to estimate the size of the ventricular system. MRI analyses were performed as previously shown, and, to avoid differences related with the brain size, the total ventricular volume was normalized with the total brain volume of each mice analyzed [13]. Briefly, animals were anesthetized using 0.5–2.5% sevoflurane with spontaneous breathing. MRI analyses were carried out using an ICON 1 Tesla system (Bruker, Billerica, MA, USA), with a mouse body radiofrequency coil. Total volume of the ventricular system and the brain were estimated from T2-weighted 3D rapid acquisition with relaxation enhancement sequences of brain coronal sections (repetition time, 95 ms; echo time, 3250 ms; plane resolution, 0.188 mm × 0.188 mm × 0.563 mm; thickness, 0.5 mm; rare factor, 8; and 32 slices). All images were analyzed with VIRTUE software (Diagnosoft, Morrisville, NC, USA) and ImageJ 1.45 software (Wayne Rasband, NIH, Bethesda, MD, USA).

### 2.4. Intraventricular Pressure Measurements

IVP was measured using a 34-gauge micropipette (Hamilton, Reno, NV, USA) filled with artificial CSF (aCSF), which was inserted into the lateral ventricle through the cerebral cortex (see Figure 3A) as previously shown [13,19]. The micropipette was coupled to a pressure transducer and communicated with a recording system (Biopac Systems, Goleta, CA, USA). Briefly, animals were anesthetized and immobilized in a stereotaxic device (Stoelting, Wood Dale, IL, USA). The micropipette, with an aCSF (120 mM NaCl, 3 mM KCl, 0.6 mM NaH_2_PO_4_, 0.8 mM MgSO_4_, 18 mM NaHCO_3_, 10mM glucose, 1.1 mM CaCl_2_; 7.4 pH) infusion rate of 0.3 μL/min [by a syringe pump (KD Scientific, Holliston, MA, USA)], was placed in the parietal cortex above the lateral ventricle (from bregma in mm: anteroposterior, −0.2; lateral, +1.0; and ventral, −1.0). When the pipette reached the parietal cortex, the pressure gradually rose. Once the pressure was >50 cm H_2_O, the infusion was stopped and the pipette was slowly advanced. The pressure fell promptly when the micropipette reached the lateral ventricle. Henceforth, IVP was recorded. A detailed example of an in vivo record of IVP, and the procedure used to infer the CSF outflow and ventricular compliance (see Section 2.5) is shown in Figure S2. IVP values were analyzed using Matlab software (Mathworks, Natic, MA, USA).

### 2.5. Cerebrospinal Fluid Outflow Dynamic and Ventricular Compliance Measurements

Dynamic of the CSF outflow was studied by the constant-rate infusion method reported before [13,19]. Concisely, a 34-gauge micropipette (Hamilton) was positioned in the lateral ventricle as described above. IVP was recorded during the continuous infusion of aCSF into the lateral ventricle (at 0.5, 1.5, 3, 7 and 14 μL/min). Each infusion rate was maintained until a stabilized IVP was recorded. That results in a step-wise increase in IVP (see example in Figure S2). The CSF outflow resistance was achieved by linear regression from the infusion rate and IVP (Figure 4A). To normalize the IVP values, the basal IVP value (obtained without aCSF infusion) were subtracted from the IVP values obtained after aCSF infusion at each rate.

Ventricular compliance was estimated by calculating the increase in IVP (∆P) produced after 1.75 μL of volume increase. This increment in the ventricular volume was induced by 15 s (t_1_ − t_0_) of aCSF infusion after increase the flow rate from 7 to 14 μL/min (see Figure 5A and Figure S2), as described previously [13].

### 2.6. Statistical Analysis

Each experimental group consisted of 7–12 animals; the specific numbers (n) are displayed in each figure legend. Data are presented as mean ± standard error of the mean (SEM), and the statistical test performed is specified in each figure legend. For all the analysis carried out, data were tested for normality (Kolmogorov–Smirnov test) and equal variance. When these properties were confirmed, analysis of variance was carried out with Tukey HSD post hoc analysis for multiple group comparisons. All statistical analyses were conducted by IBM SPSS Statistics for Windows (Version 20) or GraphPad Prism 6 packages.

## 3. Results

### 3.1. Histological Analyses of AQP1 and AQP4 Expression on the Different AQPs Knock Out Mice

**Figure 1:**
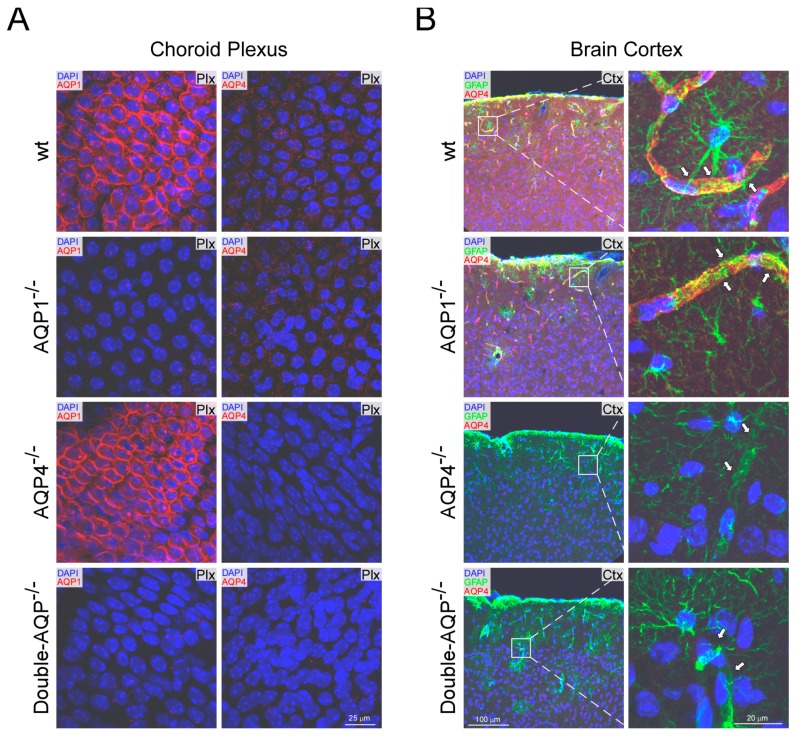
Histological analysis of AQP1 and AQP4 expression in wt and cerebral AQPs KO mice. (**A**) AQP1 and AQP4 immunofluorescence images from choroid plexus obtained from wt, AQP1^−/−^, AQP4^−/−^ and double-AQP^−/−^ KO mice. (**B**) Immunofluorescence AQP4 and GFAP images obtained from brain coronal sections of wt, AQP1^−/−^, AQP4^−/−^ and double-AQP^−/−^ KO mice, and high magnification images from the insets displayed. The arrows indicate the perivascular foot processes of GFAP^+^ astrocytes. GFAP, glial fibrillary acidic protein; DAPI, 4′,6-diamidino-2-phenylindole. The images shown are representative examples of three independent animals analyzed for each genotype.

The levels of AQP1 and AQP4 expression were analyzed by immunofluorescence in different brain areas of the four experimental mice models (wt, AQP1^−/−^, AQP4^−/−^ and double-AQP^−/−^ KO mice; Figure 1). As previously stated, expression of AQP1 was only detected in the choroid plexus [18,31]; and similar levels of AQP1 immunostaining were found in the wt and AQP4^−/−^ mice (Figure 1A), indicating the absence of any compensatory up-regulation of AQP1 in the choroid plexus from AQP4^−/−^ mice. On the other side, as expected, a complete lack of AQP1 expression was observed on the choroid plexus of AQP1^−/−^ and double-AQP^−/−^ KO mice.

The analysis of AQP4 revealed, as previously indicated, a significant expression of this protein associated to astroglial and ependymal cells [13,18]. As indicated in Figure 1B, high levels of AQP4 expression were associated to pericapillary foot processes of GFAP^+^ astrocytes in both wt and AQP1^−/−^ mice, showing both genotypes similar levels and pattern of AQP4 immunostaining. In addition, the immunofluorescence analysis of AQP4 in the choroid plexus (Figure 1B), confirmed the very low levels of AQP4 protein in this tissue, almost absent, under physiological conditions [13,18]. Moreover, a complete absence of AQP4 expression was found on AQP4^−/−^ and double-AQP^−/−^ KO mice.

The performed histological analysis confirmed the differential expression pattern of AQP1 and AQP4 in the brain [13,18], showing that AQP1 expression is restricted to epithelial cells of the choroid plexus (Figure 1), without any sign of expression in the cerebral parenchyma (Figure S3). AQP4 is highly expressed in the glia limitants (Figure 1) and ependymal cells of the cerebral parenchyma (Figure S4). However, almost negligible levels of AQP4 protein expression were found in the choroid plexus (Figure 1). In addition, no compensatory regulation of AQP1 or AQP4 in the different analyzed AQPs mutant was observed, supporting the adequacy of the mice models used to study the specific contribution of AQP1 and AQP4 to the CSF homeostasis.

### 3.2. Study of the Volume and Intraventricular Pressure in the Cerebral Ventricular System

**Figure 2:**
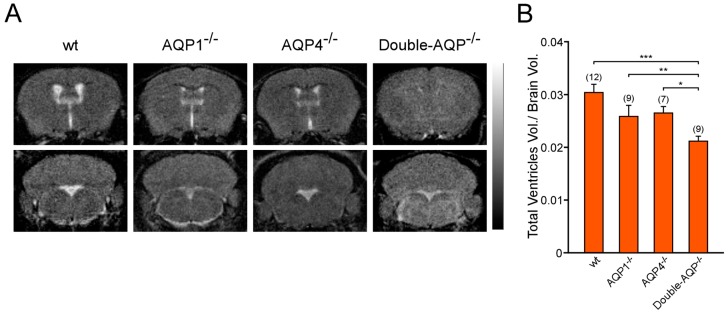
Analysis by magnetic resonance imaging of the ventricular volume of cerebral AQPs deficient mice. (**A**) 3D T2-weighted MRI of brain coronal sections from wt, AQP1^−/−^, AQP4^−/−^ and double-AQP^−/−^ KO mice. (**B**) Quantitative analysis of volume of the cerebral ventricular system, stated as the ratio of total ventricular volume/brain volume, for the four experimental groups described in A. Data are presented as mean ± S.E.M. The number of animals analyzed per experimental condition is indicated in parenthesis in the figure. * *p* < 0.05; ** *p* < 0.01; *** *p* < 0.001.

To study the specific contribution of AQP1 and AQP4 to the ventricular volume, the volume occupied for the cerebral ventricular system was assessed in wt, AQP1^−/−^, AQP4^−/−^ and double-AQP^−/−^ KO mice. Ventricle size was measured from MRI images of brain coronal sections, as an indirect indicator of CSF production, and was represented as the ratio of total ventricular volume/brain volume for all experimental groups (Figure 2A,B). As indicated in Figure 2B, AQP1^−/−^ and AQP4^−/−^ mice showed a slight reduction in the ventricle size with respect to wt controls (~15% smaller in both cases). Moreover, double-AQP^−/−^ KO mice presented an additive decrease in the ventricle size (~30% of reduction with respect to wt controls). Interestingly, the double-AQP^−/−^ KO mice presented diminished ventricle size whether compared with AQP1^−/−^ or AQP4^−/−^ mice, indicating a similar contribution of AQP1 and AQP4 to the ventricular volume.

**Figure 3:**
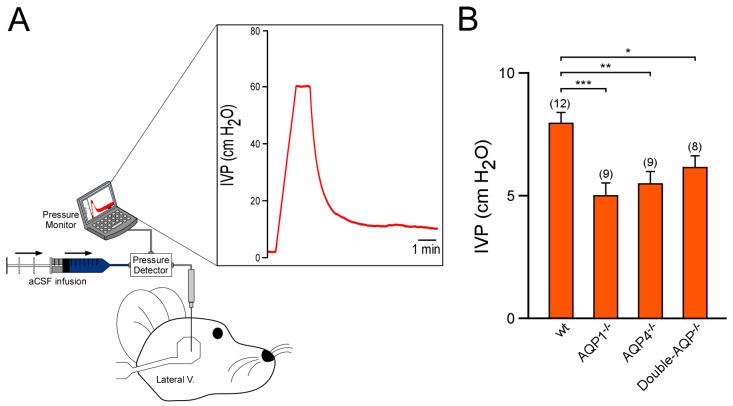
Study of the intraventricular pressure of cerebral AQPs knock out mice. (**A**) Schematic diagram showing the experimental setup used to perform the records of IVP. The graph displayed shows an example of IVP record. Briefly, the micropipette, with aCSF infusion, placed in the parietal cortex above the lateral ventricle produced a peak of pressure. Once the pressure was >50 cm H_2_O, the aCSF infusion was stopped, and the pipette was slowly lowered. The pressure drops promptly when the micropipette entered the lateral ventricle. Hereafter, the IVP was recorded. (**B**) IVP values obtained from wt, AQP1^−/−^, AQP4^−/−^ and double-AQP^−/−^ KO mice. Data are presented as mean ± S.E.M. The number of animals analyzed per experimental condition is indicated in parenthesis in the figure. * *p* < 0.05; ** *p* < 0.01; *** *p* < 0.001.

We also analyzed the IVP of the previously described experimental groups (Figure 3). As reported before, by us and other authors, AQP1^−/−^ mice showed a significant lower IVP value than wt controls (~40% smaller with respect to wt controls [13,19]). Similarly, AQP4^−/−^ mice also exhibited a clear reduction on IVP (~30% reduced with respect to wt controls), confirming the relevant participation of this protein in the CSF formation and the ventricular volume. Nevertheless, double-AQP^−/−^ KO mice did not present further lower IVP values than AQP1^−/−^ or AQP4^−/−^, which can be attributed to the extremely reduced ventricular system existing in these double mutant mice. Taken together, the MRI and IVP analysis performed in the different AQPs mutants clearly showed that both AQP1 and AQP4 contribute similarly to the volume of the brain ventricular system.

### 3.3. Analysis of CSF outflow and Ventricular Compliance

**Figure 4:**
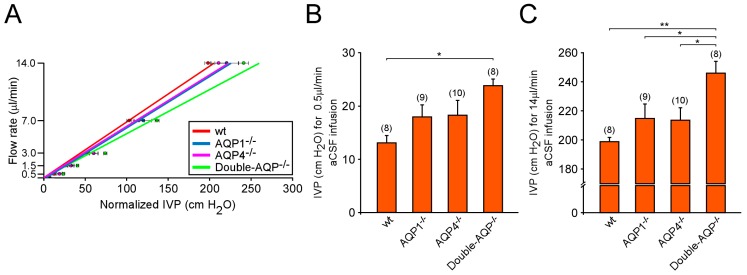
Cerebrospinal fluid outflow dynamics in AQPs deficient mice. (**A**) Pressure-dependent CSF flow rate obtained from wt, AQP1^−/−^, AQP4^−/−^ and double-AQP^−/−^ KO mice. Basal IVP values, obtained before starting aCSF infusion, were subtracted from the IVP values obtained after aCSF infusion at each rate (Normalized IVP). (**B**,**C**) Comparative analysis of the IVP reached, at steady-state, after the intraventricular infusion of aCSF at 0.5 μL/min (**B**) and 14 μL/min (**C**) on the previously described experimental groups. Data are presented as mean ± S.E.M. The number of animals analyzed per experimental condition is indicated in parenthesis in the figure. * *p* < 0.05; ** *p* < 0.01.

The participation of AQP1 and AQP4 in the CSF drainage was analyzed by experiments of pressure-dependent CSF outflow performed in wt, AQP1^−/−^, AQP4^−/−^ and double-AQP^−/−^ KO mice. In accordance with previously reported data by our group and other authors [13,19], the comparison of the slopes obtained from the linear regression between the CSF infusion rate and the IVP recorded showed no significant differences among wt, AQP1^−/−^ and AQP4^−/−^ mice (wt: 0.0689; AQP1^−/−^: 0.0609 and AQP4^−/−^: 0.0635; Figure 4A). However, the slope observed in double-AQP^−/−^ KO mice (0.0546) indicated a decreased CSF outflow in these mice with respect to wt controls. To perform a deeper analysis of the CSF drainage, we compared the IVP obtained under low (0.5 μL/min; Figure 4B) and high (14 μL/min; Figure 4C) CSF infusion rate in our experimental groups. Some alterations in the capacity to drainage the infused aCSF were observed in AQP1^−/−^ and AQP4^−/−^ mice, at low or high perfusion rate, although the differences were not statistically significant. However, the double-AQP^−/−^ KO mice showed a significant lower ability to drainage the aCSF in the two analyzed conditions (Figure 4B,C).

**Figure 5:**
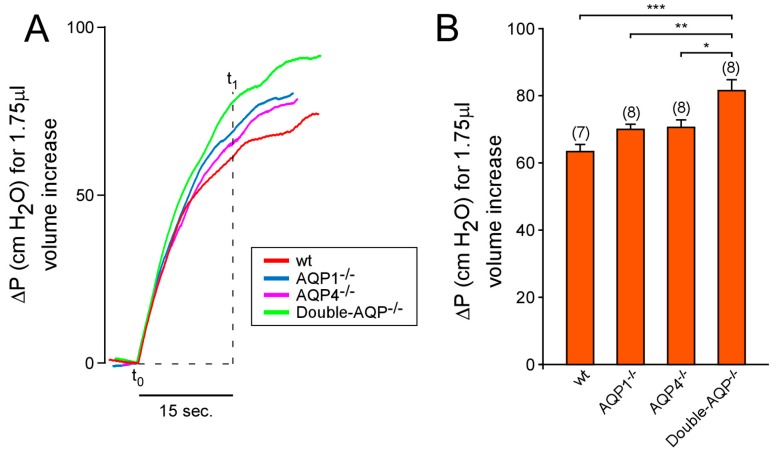
Effect of AQPs absence on the distensibility of the cerebral ventricular system. (**A**) Representative in vivo records of the increment of intraventricular pressure (ΔP) obtained after 1.75 μL of volume increase [produced by 15 seconds (t_1_ − t_0_) of aCSF infusion after increase the flow rate from 7 to 14 μL/min) on wt, AQP1^−/−^, AQP4^−/−^ and double-AQP^−/−^ KO mice. (**B**) Quantitative analysis of the ΔP in the experimental groups described before. The ventricular compliance (C) is inversely proportional to ΔP: such as C = V/ΔP. Data are presented as mean ± S.E.M. The number of animals analyzed per experimental condition is indicated in parenthesis in the figure. * *p* < 0.05; ** *p* < 0.01; *** *p* < 0.001.

We also studied the ability of the cerebral ventricular system to be distended after an increment of the CSF volume, which we called ventricular distensibility or compliance. To do that, we analyzed the increment of pressure (∆P) produced in the ventricle after 1.75 μL of aCSF volume increase (Figure 5). Similarly, as explained above for the CSF outflow, the single lack of AQP1 or AQP4 did not significantly alter the ventricular compliance. However, the simultaneous absence of both AQPs showed a significant reduction of the ventricular compliance (Figure 5A,B). Taken together, the reported data indicate that the single AQP1 or AQP4 absence is not enough to alter considerably the CSF outflow dynamics and the ventricular compliance. Nevertheless, the total deficiency of the principal brain AQPs clearly modifies these processes, suggesting the participation of AQP1 and AQP4 on them.

## 4. Discussion

AQP1 and AQP4 are the principal water-transporter channels in the central nervous system showing a distinct and complementary expression pattern within the brain. Both proteins have been proposed to be principal regulators of the cerebral fluid homeostasis in health as well as pathophysiological conditions. AQP1, exclusively expressed in the apical side of epithelial choroid plexus cells, and AQP4, mainly expressed in astrocytes and ependymal cells [18,32], have been implicated in a recent and intense debate about the CSF/ISF homeostasis. According to the “classic” conception, CSF is mainly produced in the choroid plexus where the water secretion is facilitated by AQP1 [1,4,19] and the fluid circulates unidirectionally into the subarachnoid space to be finally absorbed in the arachnoid granulations toward the dural venous sinuses. However, recent evidence supports an important extra-choroidal CSF formation, where CSF is significantly produced as consequence of water filtration from the brain parenchyma capillaries, being the water movement facilitated by AQP4 [5,20]. In the present work, by in vivo analysis performed on wt, AQP1, AQP4 and double-AQP1-AQP4 null mice, we studied the specific contribution of AQP1 and AQP4 to fundamental aspects of CSF homeostasis such as the volume of the ventricles, the CSF outflow dynamics and ventricular compliance. Regarding the CSF production and ventricle load, our MRI and IVP data clearly indicate that both AQPs participate in the CSF formation. Interestingly, AQP1^−/−^ and AQP4^−/−^ mice showed a comparable reduction in the ventricular volume and IVP suggesting that, in terms of CSF production, choroidal-AQP1 and parenchymal-AQP4 have a similar contribution to the CSF formation. In accordance with the participation of both cerebral AQPs in the ventricular volume, double-AQP^−/−^ KO mice displayed lower ventricular volume than single AQP1 or AQP4 mutants. Despite the reduction in the ventricular volume observed in double-AQP^−/−^ KO mice, with respect to AQP1^−/−^ or AQP4^−/−^ KO mice, these animals present similar IVP values to those observed in the single AQPs KO mutants. These observations suggest a functional coupling between the CSF production, facilitated either by choroidal AQP1 or astroglial and ependymal AQP4, and the ventricular volume to maintain the intracranial pressure under homeostatic values.

The study of the CSF drainage performed by pressure-dependent CSF outflow revealed that single AQP1 or AQP4 mutants do not present significant alterations in the CSF drainage. These results are in accordance with previously reported analysis performed by Verkman’s group for AQP1^−/−^ mice [19] and our group in the case of AQP4^−/−^ mice [13]. However, the results obtained in the double-AQP mutant indicate that both proteins could participate, directly or indirectly, on the CSF outflow dynamics. However additional analysis of the CSF drainage process is necessary to understand it and to identify where and how AQP1 and AQP4 might contribute to it. The potential participation of the cerebral AQPs in the CSF drainage and CSF/ISF interchange has been proposed in experimental model of hydrocephalus [33,34,35,36], but the specific mechanism by which these AQPs could facilitate the CSF drainage is still unknown. The recent description of the AQP4-mediated “glymphatic system” and its functional coupling with the meningeal lymphatics [3,6,8,37] have provided additional evidence supporting that cerebral AQPs could participate in the ISF/CSF interchange and drainage.

Regarding the contribution of AQP1 and AQP4 to the ventricular compliance, the data obtained in the double-AQP^−/−^ KO mice suggest, unexpectedly, that cerebral AQPs could regulate the ventricular compliance. The mechanism by which these AQPs could alter the ventricular system distensibility is unidentified at the present time. Additional experimental analysis should be performed to understand if the reduced ventricular volume could be related with this alteration, or if the absence of cerebral AQPs alters the biophysical properties of the cerebral parenchyma; or even if this modification could be associated with systemic alterations that potentially modify the cerebral blood flow, and secondarily alter the cerebral compliance.

In addition to AQP1 and AQP4, the aquaglyceroporin AQP9 is also expressed in the brain, being localized in astrocytes in the glial limitants, endothelial cells of the pial vessels and specific groups of neurons [38,39]. Again, the specific contribution of AQP9 to different aspects of CSF dynamics and its possible alterations on cerebral pathologies is an issue of growing interest [40,41], and might contribute to better understand yet unresolved questions, but should be further addressed with more experimental and clinical studies. In this respect, although most of the knowledge concerning the role of AQP1 and AQP4 on the CSF homeostasis has been obtained in animal models, during recent years, growing studies indicate that these AQPs might have similar functions in humans, and their alterations would been implicated in different pathophysiological conditions [7,15,42,43]. Moreover, in the last years, another AQP, AQP11, has been reported to be expressed in the brain [44,45]. Further experimental work should be performed to address if some of the modifications observed in the double-AQP^−/−^ KO could be related with compensation of other AQPs and/or alterations in the brain water content.

In conclusion, our results clearly indicate that glial- and ependymal-expressed AQP4 facilitate the CSF formation, having a quantitatively similar contribution to this process that choroidal-expressed AQP1. The fact that AQP4 facilitates the CSF production is aligned with the recently formulated “Bulat–Klarica–Oreskovic hypothesis” that predicts a significant CSF production by water filtration from the cerebral capillaries. However, the results obtained from the AQP1 mutant mice, by us and others [19], also imply a significant participation of the choroid plexus in the CSF production and IVP, contrasting with previous reports that question the participation of the choroid plexus on the CSF pressure regulation [5]. In addition, the analysis performed on the double AQP1-AQP4 null mice suggests that the principal cerebral AQPs, AQP1 and AQP4, could modify the CSF outflow dynamics and the ventricular compliance. Nevertheless, additional experimental analysis should be performed to determine the specific localization and function of these AQPs in the classic drainage routes and in the recently identified meningeal lymphatics, their relation with other brain AQPs, and how the lack of any of these cerebral AQPs could modify the structural properties of the cerebral parenchyma. All these would permit obtaining a better understanding of the precise mechanism by which the absence of AQP1 and AQP4 expression could modify these processes.

## Supplementary Materials

The following are available online https://www.mdpi.com/2073-4409/8/2/197/s1, Figure S1: General characterization of double-AQP^−/−^ mice. (A) Number of offspring, percentage and survival after birth of double-AQP^−/−^ mice (AQP1^−/−^:AQP4^−/−^). (B) Weight and brain volume of adults double-AQP^−/−^ mice, expressed as mean ± s.e.m., Figure S2: Example of the methodology used to perform the IVP records, and to infer the CSF outflow dynamics and ventricular compliance. A 34-gauge micropipette was placed in the parietal cortex above the lateral ventricle with aCSF infusion at 0.3 μL/min, which produced a gradual increase of the pressure. Once the pressure was >50 cm H_2_O, the infusion was stopped and the pipette was slowly advanced. The pressure fell promptly when the micropipette reached the lateral ventricle. Then, the IVP was recorded for 10 min (label in green). After that, the CSF outflow resistance was analyzed by the constant-rate infusion method. Briefly, IVP was recorded during the continuous infusion of aCSF into the lateral ventricle (at 0.5, 1.5, 3, 7 and 14 μL/min). Each infusion rate was maintained until a stabilized IVP was recorded, which results in a step-wise increase in IVP (label in blue). Finally, the ventricular compliance was estimated by calculating the increase in IVP (∆P) produced after 1.75 μL of volume increase (label in red); this increment in the ventricular volume was induced by 15 s (t_1_ − t_0_) of aCSF infusion after increase the flow rate from 7 to 14 μL/min, Figure S3: Absence of AQP1 expression in brain parenchyma. Immunofluorescence AQP1 and GFAP images of the brain cortex, obtained from brain coronal sections of wt, AQP1^−/−^, AQP4^−/−^ and double-AQP^−/−^ KO mice, illustrating the lack of AQP1 expression in the cerebral parenchyma. GFAP: glial fibrillary acidic protein. DAPI: 4′,6-diamidino-2-phenylindole. The images shown are representative examples of three independent animals analyzed for each genotype, Figure S4: AQP4 expression in lateral ventricles. Immunofluorescence AQP4 and GFAP images of the lateral ventricles, obtained from brain coronal sections of wt, AQP1^−/−^, AQP4^−/−^ and double-AQP^−/−^ KO mice. GFAP: glial fibrillary acidic protein. DAPI: 4′,6-diamidino-2-phenylindole. The images shown are representative examples of three independent animals analyzed for each genotype.

## References

[1] Johanson C.E., Duncan J.A., Klinge P.M., Brinker T., Stopa E.G., Silverberg G.D. (2008). Multiplicity of cerebrospinal fluid functions: New challenges in health and disease. Cerebrospinal Fluid Res..

[2] Abbott N.J., Pizzo M.E., Preston J.E., Janigro D., Thorne R.G. (2018). The role of brain barriers in fluid movement in the CNS: Is there a ‘glymphatic’ system?. Acta Neuropathol..

[3] Da Mesquita S., Fu Z., Kipnis J. (2018). The Meningeal Lymphatic System: A New Player in Neurophysiology. Neuron.

[4] Hladky S.B., Barrand M.A. (2014). Mechanisms of fluid movement into, through and out of the brain: Evaluation of the evidence. Fluids Barriers CNS.

[5] Oreskovic D., Rados M., Klarica M. (2017). Role of choroid plexus in cerebrospinal fluid hydrodynamics. Neuroscience.

[6] Iliff J.J., Wang M., Liao Y., Plogg B.A., Peng W., Gundersen G.A., Benveniste H., Vates G.E., Deane R., Goldman S.A. (2012). A paravascular pathway facilitates CSF flow through the brain parenchyma and the clearance of interstitial solutes, including amyloid beta. Sci. Transl. Med..

[7] Ringstad G., Vatnehol S.A.S., Eide P.K. (2017). Glymphatic MRI in idiopathic normal pressure hydrocephalus. Brain.

[8] Benveniste H., Lee H., Volkow N.D. (2017). The Glymphatic Pathway: Waste Removal from the CNS via Cerebrospinal Fluid Transport. Neuroscientist.

[9] Plog B.A., Nedergaard M. (2018). The Glymphatic System in Central Nervous System Health and Disease: Past, Present, and Future. Annu. Rev. Pathol..

[10] Louveau A., Smirnov I., Keyes T.J., Eccles J.D., Rouhani S.J., Peske J.D., Derecki N.C., Castle D., Mandell J.W., Lee K.S. (2015). Structural and functional features of central nervous system lymphatic vessels. Nature.

[11] Aspelund A., Antila S., Proulx S.T., Karlsen T.V., Karaman S., Detmar M., Wiig H., Alitalo K. (2015). A dural lymphatic vascular system that drains brain interstitial fluid and macromolecules. J. Exp. Med..

[12] Xiang J., Routhe L.J., Wilkinson D.A., Hua Y., Moos T., Xi G., Keep R.F. (2017). The choroid plexus as a site of damage in hemorrhagic and ischemic stroke and its role in responding to injury. Fluids Barriers CNS.

[13] Trillo-Contreras J.L., Ramirez-Lorca R., Hiraldo-Gonzalez L., Sanchez-Gomar I., Galan-Cobo A., Suarez-Luna N., Sanchez de Rojas-de Pedro E., Toledo-Aral J.J., Villadiego J., Echevarria M. (2018). Combined effects of aquaporin-4 and hypoxia produce age-related hydrocephalus. Biochim. Biophys. Acta Mol. Basis Dis..

[14] Silverberg G., Mayo M., Saul T., Rubenstein E., McGuire D. (2003). Alzheimer’s disease, normal-pressure hydrocephalus, and senescent changes in CSF circulatory physiology: A hypothesis. Lancet Neurol..

[15] Suzuki Y., Nakamura Y., Yamada K., Igarashi H., Kasuga K., Yokoyama Y., Ikeuchi T., Nishizawa M., Kwee I.L., Nakada T. (2015). Reduced CSF Water Influx in Alzheimer’s Disease Supporting the beta-Amyloid Clearance Hypothesis. PLoS ONE.

[16] Tarasoff-Conway J.M., Carare R.O., Osorio R.S., Glodzik L., Butler T., Fieremans E., Axel L., Rusinek H., Nicholson C., Zlokovic B.V. (2015). Clearance systems in the brain-implications for Alzheimer disease. Nat. Rev. Neurol..

[17] Amiry-Moghaddam M., Ottersen O.P. (2003). The molecular basis of water transport in the brain. Nat. Rev. Neurosci..

[18] Papadopoulos M.C., Verkman A.S. (2013). Aquaporin water channels in the nervous system. Nat. Rev. Neurosci..

[19] Oshio K., Watanabe H., Song Y., Verkman A.S., Manley G.T. (2005). Reduced cerebrospinal fluid production and intracranial pressure in mice lacking choroid plexus water channel Aquaporin-1. FASEB J..

[20] Igarashi H., Tsujita M., Kwee I.L., Nakada T. (2014). Water influx into cerebrospinal fluid is primarily controlled by aquaporin-4, not by aquaporin-1: 17O JJVCPE MRI study in knockout mice. Neuroreport.

[21] Jessen N.A., Munk A.S., Lundgaard I., Nedergaard M. (2015). The Glymphatic System: A Beginner’s Guide. Neurochem. Res..

[22] Smith A.J., Yao X., Dix J.A., Jin B.J., Verkman A.S. (2017). Test of the ‘glymphatic’ hypothesis demonstrates diffusive and aquaporin-4-independent solute transport in rodent brain parenchyma. eLife.

[23] Smith A.J., Verkman A.S. (2018). The “sglymphatic”s mechanism for solute clearance in Alzheimer’s disease: Game changer or unproven speculation?. FASEB J..

[24] Song Y., Ma T., Matthay M.A., Verkman A.S. (2000). Role of aquaporin-4 in airspace-to-capillary water permeability in intact mouse lung measured by a novel gravimetric method. J. Gen. Physiol..

[25] Ma T., Yang B., Gillespie A., Carlson E.J., Epstein C.J., Verkman A.S. (1998). Severely impaired urinary concentrating ability in transgenic mice lacking aquaporin-1 water channels. J. Biol. Chem..

[26] Muñoz-Cabello A.M., Villadiego J., Toledo-Aral J.J., López-Barneo J., Echevarría M. (2010). AQP1 mediates water transport in the carotid body. Pflügers Arch. Eur. J. Physiol..

[27] Galan-Cobo A., Ramirez-Lorca R., Echevarria M. (2016). Role of aquaporins in cell proliferation: What else beyond water permeability?. Channels (Austin).

[28] Villadiego J., Labrador-Garrido A., Franco J.M., Leal-Lasarte M., De Genst E.J., Dobson C.M., Pozo D., Toledo-Aral J.J., Roodveldt C. (2018). Immunization with alpha-synuclein/Grp94 reshapes peripheral immunity and suppresses microgliosis in a chronic Parkinsonism model. Glia.

[29] Villadiego J., Romo-Madero S., Garcia-Swinburn R., Suarez-Luna N., Bermejo-Navas A., Echevarria M., Toledo-Aral J.J. (2018). Long-term immunosuppression for CNS mouse xenotransplantation: Effects on nigrostriatal neurodegeneration and neuroprotective carotid body cell therapy. Xenotransplantation.

[30] Munoz-Manchado A.B., Villadiego J., Suarez-Luna N., Bermejo-Navas A., Garrido-Gil P., Labandeira-Garcia J.L., Echevarria M., Lopez-Barneo J., Toledo-Aral J.J. (2013). Neuroprotective and reparative effects of carotid body grafts in a chronic MPTP model of Parkinson’s disease. Neurobiol. Aging.

[31] Nielsen S., Smith B.L., Christensen E.I., Agre P. (1993). Distribution of the aquaporin CHIP in secretory and resorptive epithelia and capillary endothelia. Proc. Natl. Acad. Sci. USA.

[32] Nagelhus E.A., Ottersen O.P. (2013). Physiological roles of aquaporin-4 in brain. Physiol. Rev..

[33] Bloch O., Auguste K.I., Manley G.T., Verkman A.S. (2006). Accelerated progression of kaolin-induced hydrocephalus in aquaporin-4-deficient mice. J. Cereb. Blood Flow Metab..

[34] Paul L., Madan M., Rammling M., Chigurupati S., Chan S.L., Pattisapu J.V. (2011). Expression of aquaporin 1 and 4 in a congenital hydrocephalus rat model. Neurosurgery.

[35] Kalani M.Y., Filippidis A.S., Rekate H.L. (2012). Hydrocephalus and aquaporins: The role of aquaporin-1. Acta Neurochir. Suppl..

[36] Filippidis A.S., Kalani M.Y., Rekate H.L. (2012). Hydrocephalus and aquaporins: The role of aquaporin-4. Acta Neurochir. Suppl..

[37] Cao X., Xu H., Feng W., Su D., Xiao M. (2018). Deletion of aquaporin-4 aggravates brain pathology after blocking of the meningeal lymphatic drainage. Brain Res. Bull..

[38] Badaut J., Petit J.M., Brunet J.F., Magistretti P.J., Charriaut-Marlangue C., Regli L. (2004). Distribution of Aquaporin 9 in the adult rat brain: Preferential expression in catecholaminergic neurons and in glial cells. Neuroscience.

[39] Badaut P.J., Regli L. (2004). Distribution and possible roles of aquaporin 9 in the brain. Neuroscience.

[40] Badaut J. (2010). Aquaglyceroporin 9 in brain pathologies. Neuroscience.

[41] Liu H., Yang M., Qiu G.P., Zhuo F., Yu W.H., Sun S.Q., Xiu Y. (2012). Aquaporin 9 in rat brain after severe traumatic brain injury. Arq. Neuropsiquiatr..

[42] Suzuki Y., Nakamura Y., Yamada K., Huber V.J., Tsujita M., Nakada T. (2013). Aquaporin-4 positron emission tomography imaging of the human brain: First report. J. Neuroimaging.

[43] Zeppenfeld D.M., Simon M., Haswell J.D., D’Abreo D., Murchison C., Quinn J.F., Grafe M.R., Woltjer R.L., Kaye J., Iliff J.J. (2017). Association of Perivascular Localization of Aquaporin-4 With Cognition and Alzheimer Disease in Aging Brains. JAMA Neurol..

[44] Koike S., Tanaka Y., Matsuzaki T., Morishita Y., Ishibashi K. (2016). Aquaporin-11 (AQP11) Expression in the Mouse Brain. Int. J. Mol. Sci..

[45] Xi T., Jin F., Zhu Y., Wang J., Tang L., Wang Y., Liebeskind D.S., Scalzo F., He Z. (2018). miR-27a-3p protects against blood-brain barrier disruption and brain injury after intracerebral hemorrhage by targeting endothelial aquaporin-11. J. Biol. Chem..

