# Supplementary material for: AQP1 and AQP4 Contribution to Cerebrospinal Fluid Homeostasis
Source: Cells. 2019 Feb 24;8(2):197. doi: 10.3390/cells8020197 (PMC6406452; doi:10.3390/cells8020197)
Supplement: Supplementary file 1 [file cells-08-00197-s001.zip › Supp Figures/Supp-Fig1.pdf]

A

Breeding	Number of offsprings	Double-AQP$^{-/-}$ offsprings (frequency, %)	Expected frequency (%)
AQP1$^{+/-}$:AQP4$^{+/-}$ x AQP1$^{+/-}$:AQP4$^{+/-}$	110	5 **(4,54%)**	**6,25%**
AQP1$^{-/-}$:AQP4$^{+/-}$ x AQP1$^{+/-}$:AQP4$^{+/-}$	25	3 **(12,0%)**	**12,5%**
AQP1$^{-/-}$:AQP4$^{+/-}$ x AQP1$^{-/-}$:AQP4$^{+/-}$	124	33 **(26,6%)**	**25,0%**
AQP1$^{+/-}$:AQP4$^{-/-}$ x AQP1$^{+/-}$:AQP4$^{-/-}$	62	14 **(22,5%)**	**25,0%**
AQP1$^{-/-}$:AQP4$^{+/-}$ x AQP1$^{-/-}$:AQP4$^{-/-}$	25	10 **(40,0%)**	**50,0%**
AQP1$^{+/-}$:AQP4$^{-/-}$ x AQP1$^{-/-}$:AQP4$^{-/-}$	8	5 **(62,5%)**	**50,0%**
Survival after birth of Double-AQP$^{-/-}$ mice (%)		62 / 70 **(88,6%)**	

B

Mice Genotype	n	Body Weight (g)	n	Whole Brain Volume (mm^3)
wildtype (wt)	12	28,0 ± 0,52	12	404,8 ± 7,44
AQP1$^{-/-}$	10	27,4 ± 0,83	9	384,4 ± 6,03
AQP4$^{-/-}$	10	26,0 ± 1,13	7	434,7 ± 7,52
Double-AQP$^{-/-}$	11	29,9 ± 0,68	9	434,3 ± 6,47
