# Supplementary figures and images for: AQP1 and AQP4 Contribution to Cerebrospinal Fluid Homeostasis
Source: Cells. 2019 Feb 24;8(2):197. doi: 10.3390/cells8020197 (PMC6406452; doi:10.3390/cells8020197)

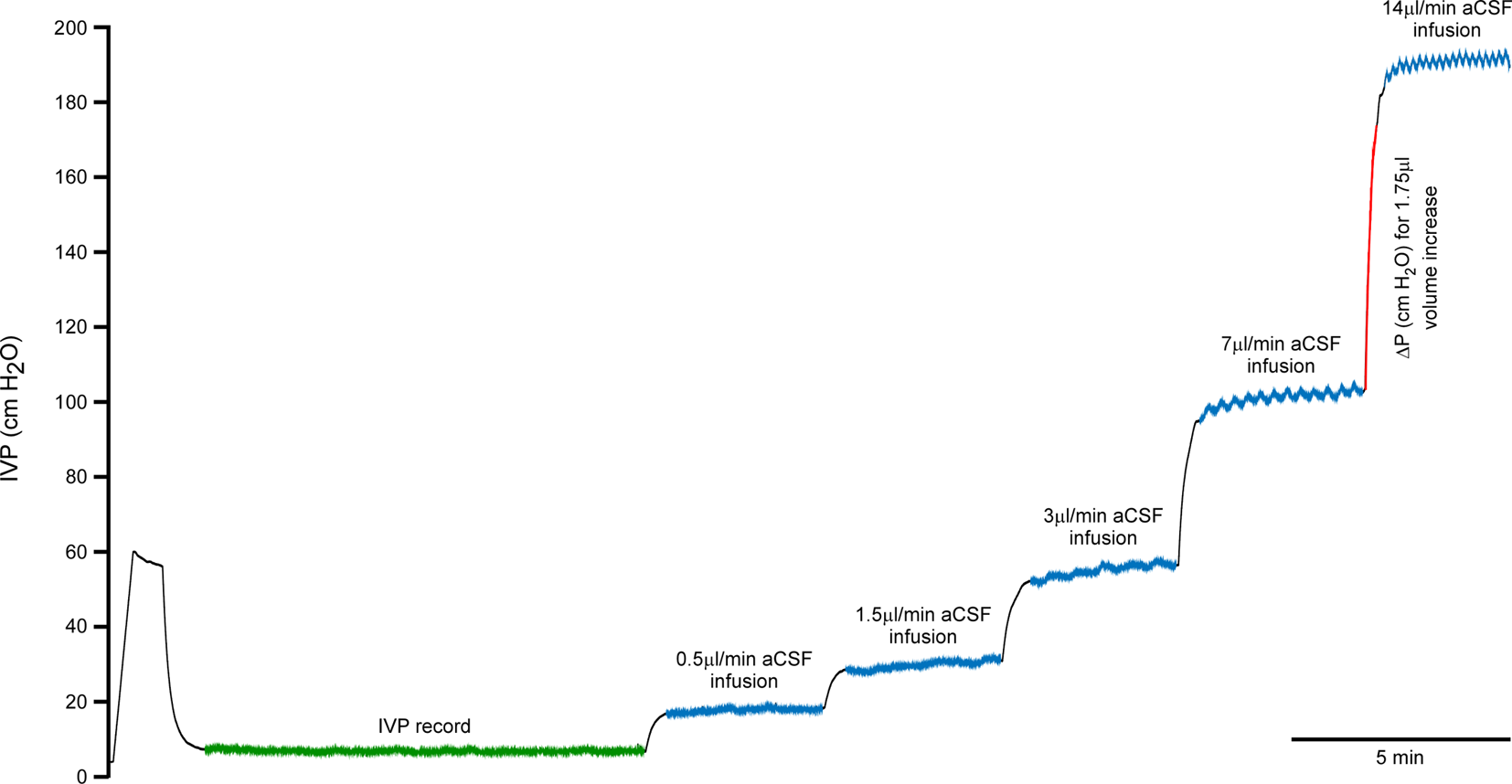

Supplement: Supplementary file 1 [file cells-08-00197-s001.zip › Supp Figures/Supp-Fig2.tif]

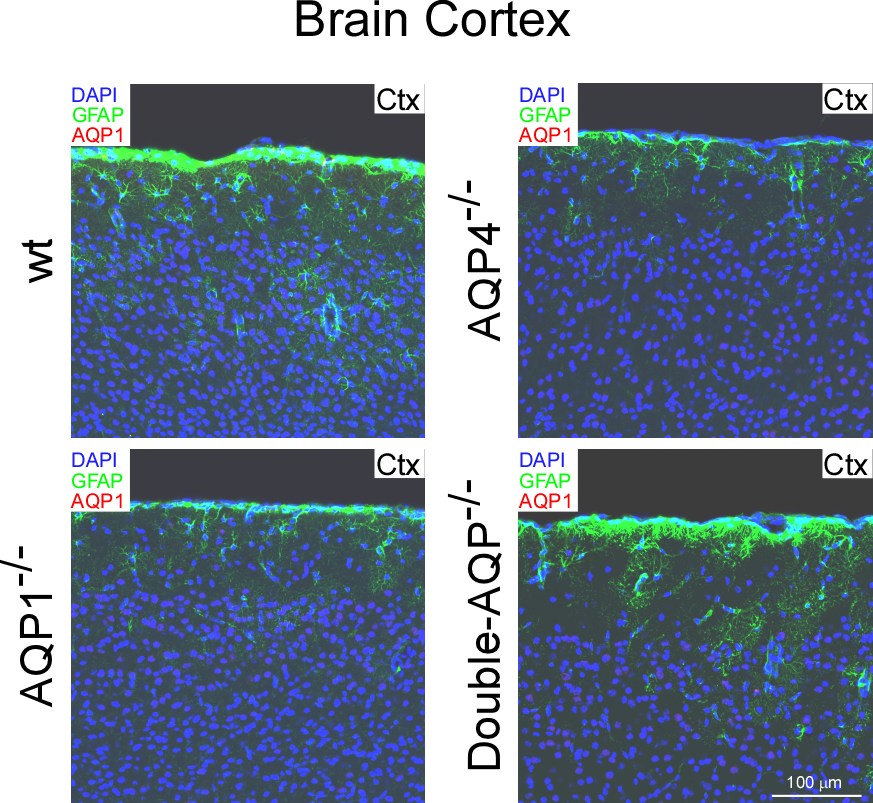

Supplement: Supplementary file 1 [file cells-08-00197-s001.zip › Supp Figures/Supp-Fig3.tif]

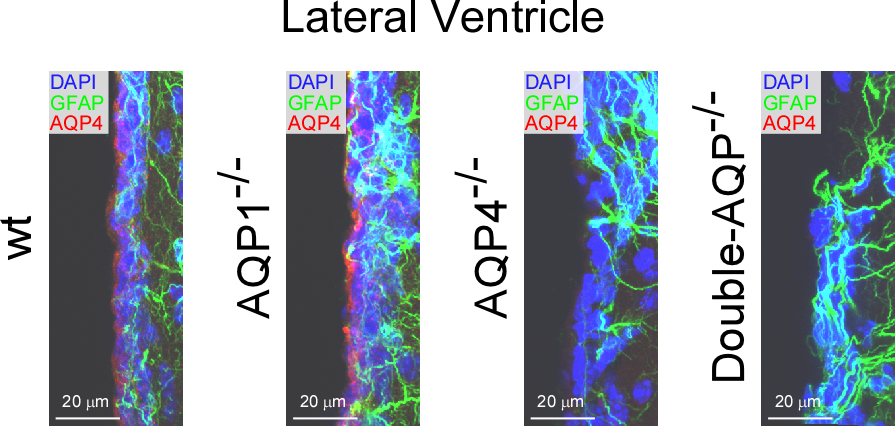

Supplement: Supplementary file 1 [file cells-08-00197-s001.zip › Supp Figures/Supp-Fig4.tif]
